# Supplementary material for: Tuneable ion selectivity in vermiculite membranes intercalated with unexchangeable ions
Source: Nat Commun. 2025 Dec 2;17:25. doi: 10.1038/s41467-025-66545-7 (PMC12764551; doi:10.1038/s41467-025-66545-7)
Supplement: Supplementary file 1 — Supplementary Information [file 41467_2025_66545_MOESM1_ESM.pdf]

Supplementary Information

Tuneable ion selectivity in vermiculite membranes intercalated with unexchangeable ions

Zhuang Liu[1,2,+,*], Yumei Tan[1,+], Jianhao Qian[3,4,+], Min Cao[1], Eli Hoenig[5], Guowei Yang[6], Fengchao Wang[3,*], Francois M. Peeters[7,8], Yi-Chao Zou[6,*], Liang-Yin Chu[1,2,*], Marcelo Lozada-Hidalgo[5,*]

[1]School of Chemical Engineering, Sichuan University, Chengdu, Sichuan 610065, P. R. China.
[2] National Key Laboratory of Advanced Polymer Materials, Sichuan University, Chengdu, Sichuan 610065, P. R. China.
[3]Department of Modern Mechanics, University of Science and Technology of China, Hefei 230027, China.
[4]Department of Civil and Environmental Engineering, Rice University, Houston, TX, 77005, USA
[5]Department of Physics and Astronomy, The University of Manchester, Manchester, M13 9PL, UK.
[6]School of Materials Science and Engineering, Sun Yat-sen University, Guangzhou, 510275, P. R. China.
[7]Departamento de Fisica, Universidade Federal do Ceara, 60455-760, Brazil.
[8]Department Physics, University of Antwerp, Groenenborgerlaan 171, B-2020 Antwerpen, Belgium.

[+]These authors contributed equally to this work.

[*]Corresponding authors: liuz@scu.edu.cn; wangfc@ustc.edu.cn; zouych5@mail.sysu.edu.cn; chuly@scu.edu.cn; marcelo.lozadahidalgo@manchester.ac.uk.

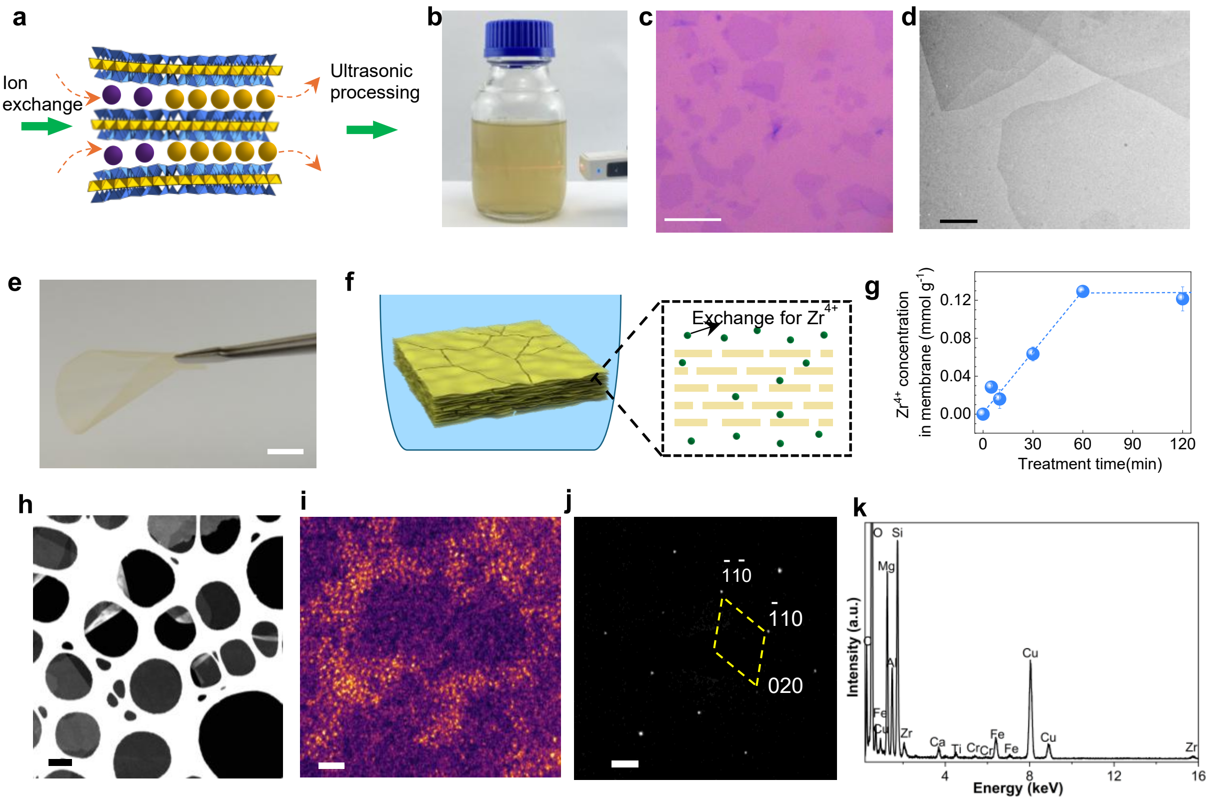

Supplementary Fig. 1 | Sample preparation. **a**, Schematic illustration of the ion exchange and exfoliation process. **b**, Vermiculite dispersions show Tyndall effect. **c**, Optical image of vermiculite flakes dispersed on silicon-dioxide substrate. Typical flake size is about 10 μm. Scale bar 10 μm. **d**, TEM images reveal that vermiculite flakes display negligible defects. Scale bar 1 μm. **e**, Optical image of free-standing vermiculite membrane. Scale bar 1 cm. **f**, Schematic showing that the membranes are soaked in different ion solutions for ion exchange. **g**, The Zr^{4+} concentration in a vermiculite laminate membrane as a function of time immersed in 1M ZrCl electrolyte solution. **h**, Image of Zr-Ver nanosheets on TEM grids. Scale bar 1 μm. **i**, STEM image of Zr-Ver over larger areas. Bright spots, Zr-atoms. Scale bar, 1 nm. **j**, Electron diffraction taken from the sample. Scale bar, 1 nm^{-1}. **k**, Corresponding EDS spectrum. Cu peaks are from the TEM holder and the supporting grids.

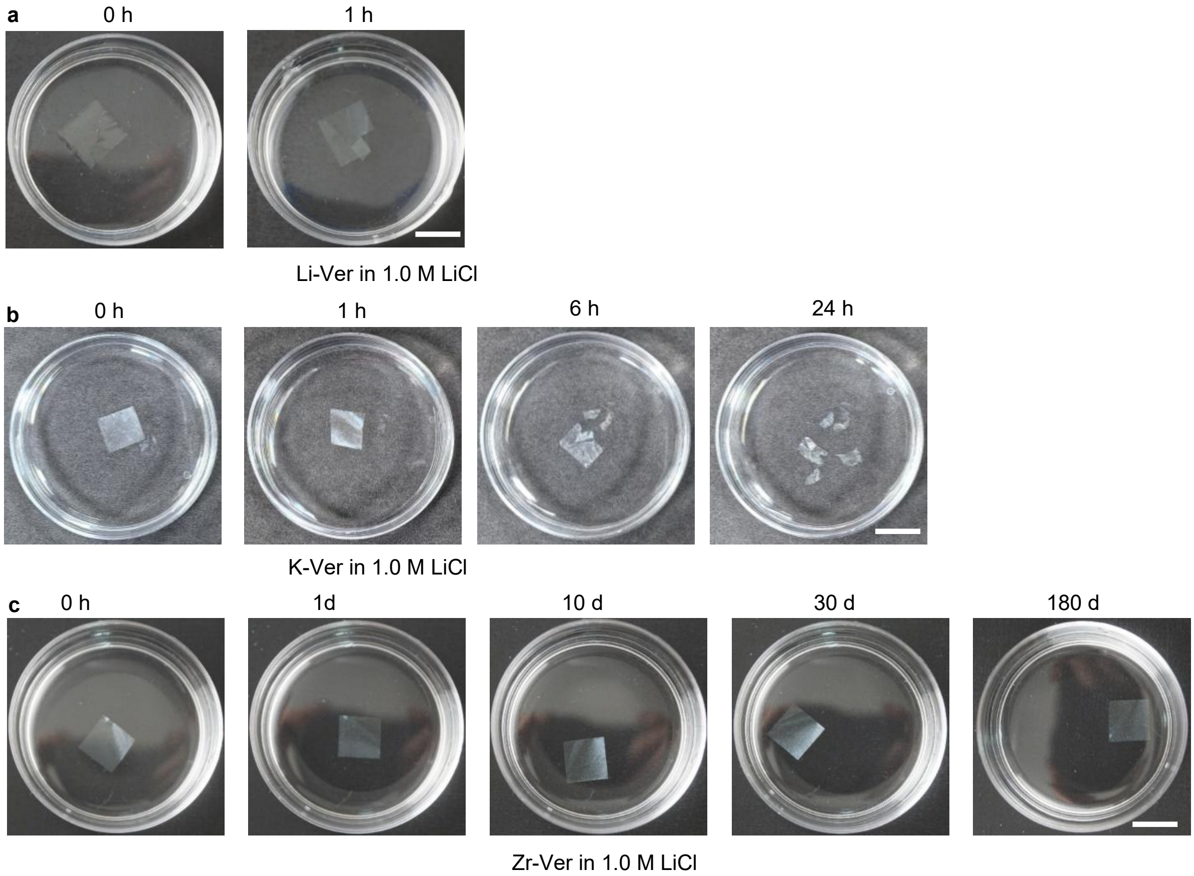

Supplementary Fig. 2| Membrane integrity in electrolyte solutions. Optical image of a Li-Ver **(a)**, K-Ver **(b)** and Zr-Ver **(c)** membrane in 1M LiCl solution, respectively. The K-Ver and Zr-Ver membranes maintain their integrity over a long period of immersion in 1M LiCl solution, with no significant damage observed macroscopically. All electrolytes tested in this work led to similar results.

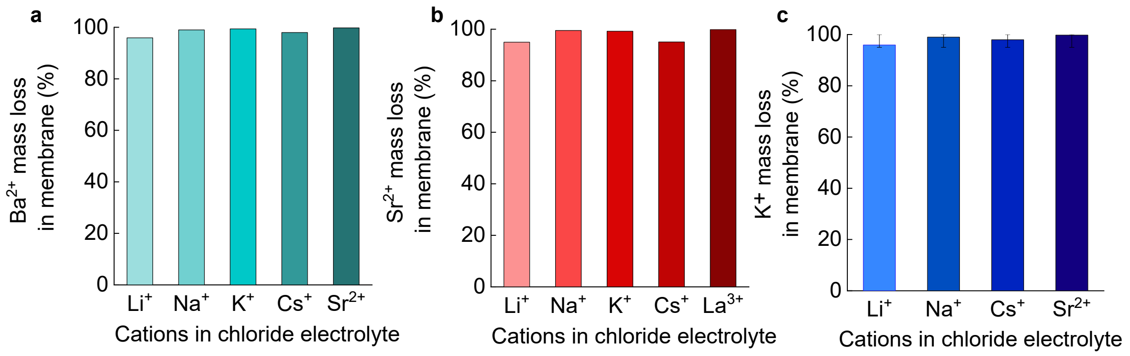

Supplementary Fig. 3 | Membranes intercalated with common ions are unstable against ion exchange. Ba^{2+}-Ver **(a)**, Sr^{2+}-Ver **(b)** and K^{+}-Ver **(c)** membranes were immersed in 1M LiCl, NaCl, KCl and SrCl electrolyte solutions for 24 hrs, respectively. The mass loss of the original intercalated Ba^{2+}, Sr^{2+} and K^{2+} measured by mass spectrometry.

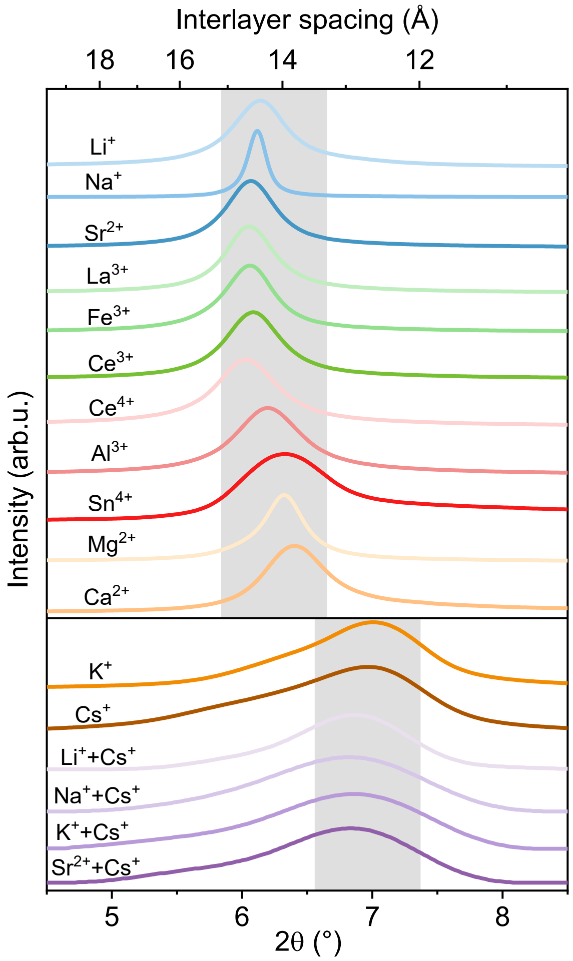

Supplementary Fig. 4 | XRD spectra of Zr-Ver membranes. XRD spectra of Zr-Ver membranes after 24 hr of immersion in 1M electrolyte solutions. The greyed area shows that the XRD peaks 2θ angles fall into two groups.

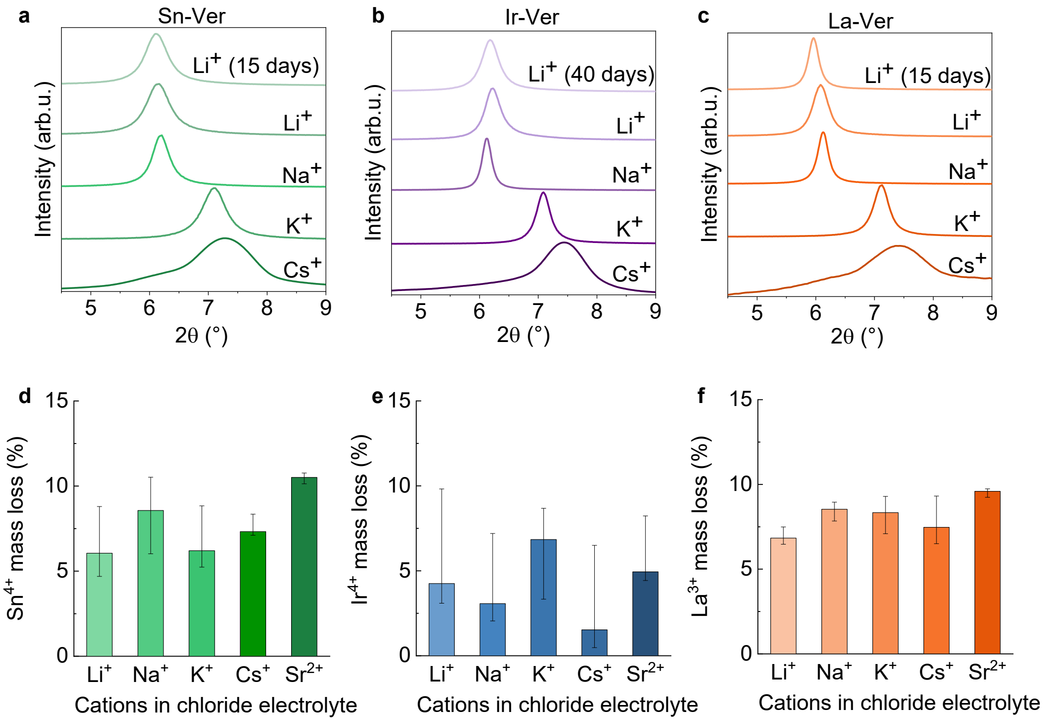

Supplementary Fig. 5 | Vermiculite membranes exchanged with unexchangeable ions are stable against re-exchange. XRD spectra of Sn-Ver **(a)**, Ir-Ver **(b)**, and La-Ver **(c)** membranes after immersion in 1M chloride salt solutions for 24 hours. **d-f**, Mass loss of intercalated unexchangeable ions in the membranes in panels **a-c**.

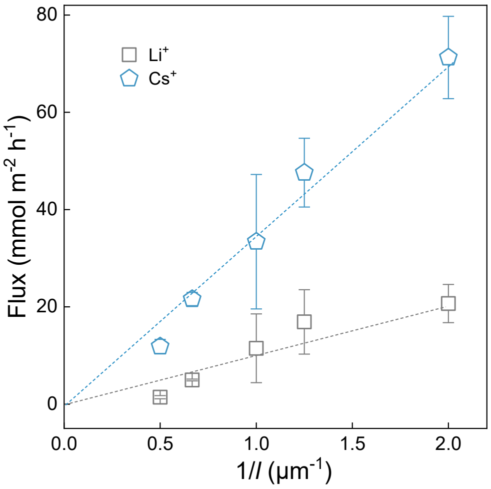

Supplementary Fig. 6 | Ion flux dependence on membrane thickness. Ion flux through Zr-Ver membranes of various thicknesses, *l*. The flux scales linearly with 1/*l* (marked with dashed lines).

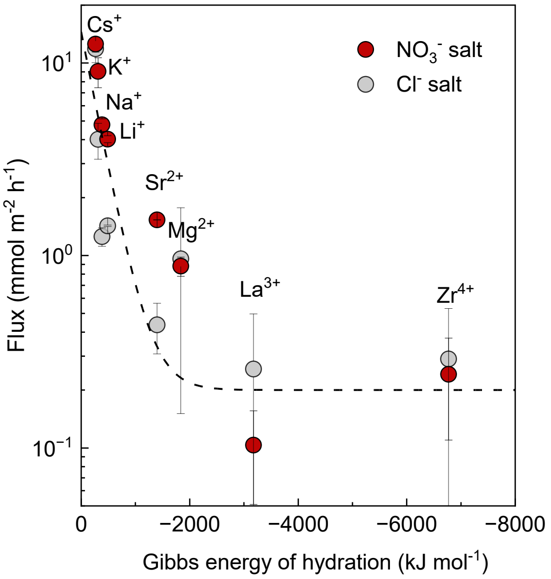

Supplementary Fig. 7 | Cation flux with nitrate and chloride salts. Ion flux vs. Gibbs hydration energy for permeating cations from nitrate (red) and chloride (grey) salts. Dashed curve, guide to the eye.

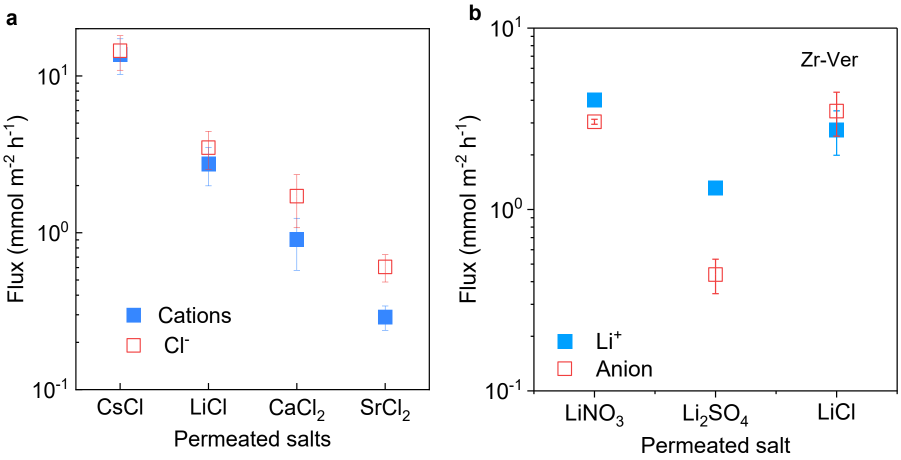

Supplementary Fig. 8 | Total ion flux through the membranes is electroneutral. a, Flux of chlorine salts with different cations. **b**, Flux of lithium salts with different anions through Zr-Ver membranes. In all cases, cation and anion transport balance to maintain charge neutrality. Strongly hydrated SO_4^{2-} permeates more slowly than Cl^-, reducing the accompanying Li^+ flux. The less strongly hydrated NO_3^- permeates at rates comparable to Cl^-, with a similar Li^+ flux.

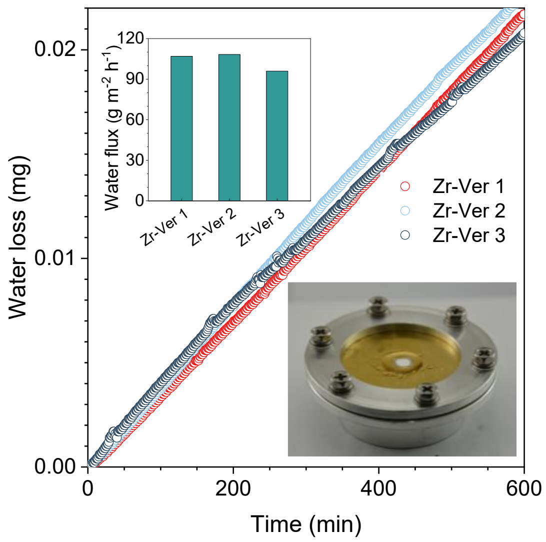

Supplementary Fig. 9 | Water vapour transport through vermiculite membranes. Water loss from a sealed container capped with different membranes (colour coded), measured gravimetrically over time. Top inset, water flux extracted from the slopes for each membrane. Bottom inset, schematic of the experimental setup.

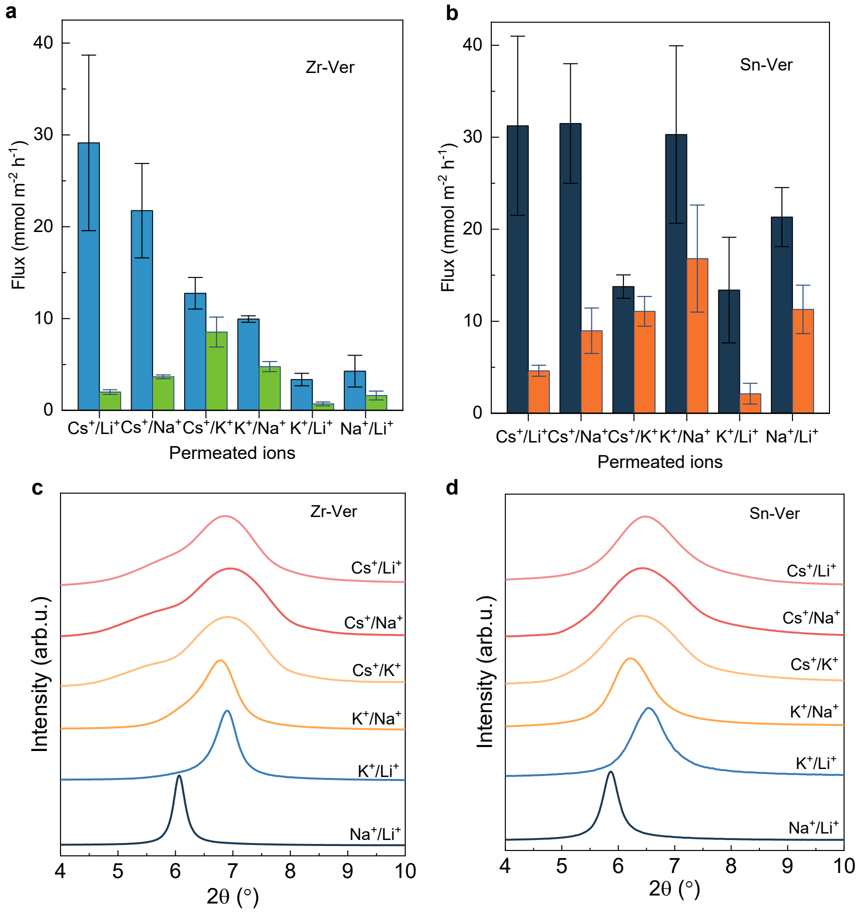

Supplementary Fig. 10 | Mixed salt transport through vermiculite membranes. a, Ion flux through Zr–Ver membranes for different monovalent ion mixtures (ion pairs shown in blue and green bars). **b**, Corresponding data for Sn–Ver membranes (ion pairs shown in blue and orange). Error bars in **a** and **b**, SD from different membranes. **c**, XRD patterns of Zr–Ver membranes after exposure to the ion mixtures. **d**, Equivalent XRD data for Sn–Ver membranes.

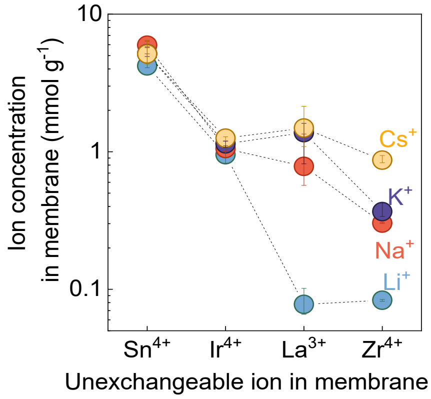

Supplementary Fig. 11 | Concentration of permeating ion inside membranes intercalated with different unexchangeable ions. Concentration of different ions (colour coded) inside Zr-Ver, La-Ver, Ir-Ver and Sn-Ver membranes after ion transport measurements, as determined by ICP-AES measurements. The concentration obtained from ICP is converted into ion concentration per channel volume ('Measurement of ion flux' in Methods). Dashed lines, guide to the eye.

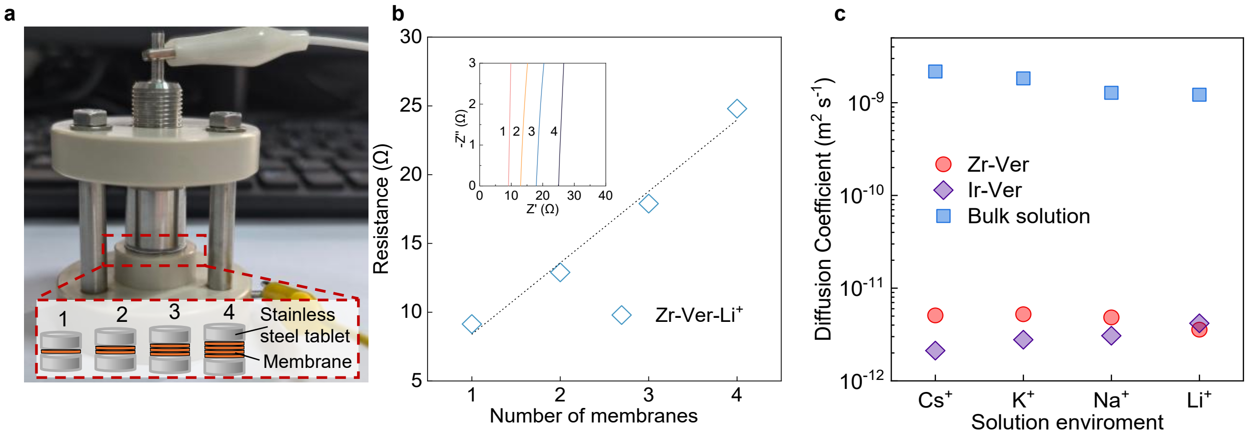

Supplementary Fig. 12 | Electrochemical impedance spectroscopy measurements. a, Experimental setup. **b**, Resistance of typical membrane stack (Zr-Ver) soaked in electrolyte solution (LiCl) as a function of number of stacked membranes. The membrane resistance was extracted from the slope of the linear fit of the resistance vs. the numbers of membranes stacked layer-by-layer. Inset, Nyquist plot of membrane stacks from which resistance in main panel is extracted. **c**, Diffusion coefficients for different ions in Zr-Ver, Ir-Ver and PES-membrane (bulk) membranes extracted from resistance data.

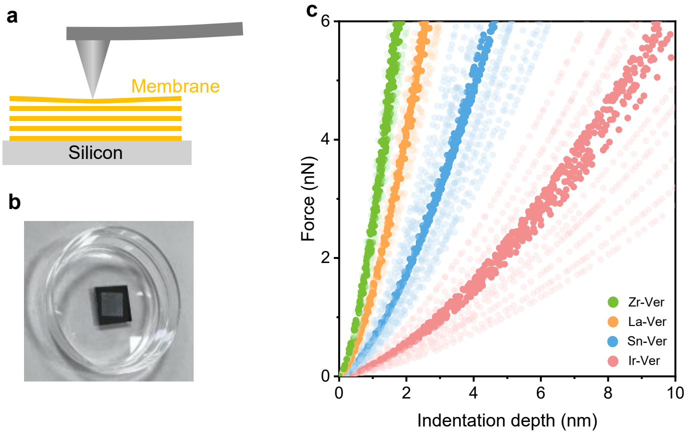

Supplementary Fig. 13 | Young's moduli of vermiculite membranes. a, Experimental setup. **b,** Optical image of vermiculite membrane on silicon substrate in liquid. **c,** Force curves from indentation experiments. Each curve was taken at a different point in the sample. Different colours indicate membranes intercalated with different unexchangeable ions. Dark (faded) coloured curves, median (typical) indentation curve for each membrane type.

Supplementary Table 1 | Ion permeabilities, membrane ion concentrations, and associated interlayer spacings

	Membrane	Ion	$C_{\text{ion in channel}}$ (mol/cm^3)	Interlayer spacings (Å)	Average flux (mmol m^2 h^{-1})	Permeability (m^2 s^{-1})
Without Cs^+	Zr-Ver	Li^+	2.45	14.8	1.43	7.92×10^{-13}
		Na^+	4.30	14.8	1.25	6.96×10^{-13}
		K^+	6.23	12.2	4.01	2.23×10^{-12}
		Cs^+	10.34	12.2	11.86	6.59×10^{-12}
		Mg^{2+}	\	14.0	0.96	5.34×10^{-13}
		Ca^{2+}	\	14.0	1.32	7.34×10^{-13}
		Sr^{2+}	\	14.8	0.44	2.42×10^{-13}
		La^{3+}	\	14.8	0.26	1.43×10^{-13}
		Zr^{4+}	\	14.8	0.29	1.61×10^{-13}
With Cs^+	Zr-Ver	Li^+	\	12.8	1.99	1.11×10^{-12}
		Na^+	\	12.8	3.66	2.03×10^{-12}
		K^+	\	12.8	8.53	4.74×10^{-12}
		Cs^+	\	\	10.50	5.83×10^{-12}
		Mg^{2+}	\	\	0.027	1.52×10^{-14}
		Ca^{2+}	\	\	0.051	2.83×10^{-14}
		Sr^{2+}	\	12.8	0.032	1.76×10^{-14}
		Ce^{3+}	\	\	0.0005	2.78×10^{-16}
		Fe^{3+}	\	\	0.0005	2.78×10^{-16}
		Al^{3+}	\	\	0.0005	2.78×10^{-16}
		La^{3+}	\	\	0.00037	2.04×10^{-16}
		Ce^{4+}	\	\	0.0001	5.56×10^{-17}

		Sn^{4+}	\	\	0.0001	5.56×10^{-17}
		Zr^{4+}	\	\	0.0003	1.67×10^{-16}
Without Cs^{+}	La-Ver	Li^{+}	1.93	14.5	0.89	4.94×10^{-13}
		Na^{+}	8.14	14.4	5.70	3.17×10^{-12}
		K^{+}	16.10	12.2	5.61	3.12×10^{-12}
		Cs^{+}	22.78	11.9	8.40	4.67×10^{-12}
Without Cs^{+}	Ir-Ver	Li^{+}	7.86	14.2	85.30	4.74×10^{-11}
		Na^{+}	8.56	14.4	14.08	7.82×10^{-12}
		K^{+}	14.99	12.3	11.81	6.56×10^{-12}
		Cs^{+}	20.04	11.9	13.27	7.37×10^{-12}
Without Cs^{+}	Sn-Ver	Li^{+}	36.22	14.3	91.42	5.08×10^{-11}
		Na^{+}	50.18	14.3	12.41	6.89×10^{-12}
		K^{+}	65.36	12.3	17.60	9.78×10^{-12}
		Cs^{+}	73.10	12.0	21.84	1.21×10^{-11}
